# Re-emergence of the leaf clip gesture during an alpha takeover affects variation in male chimpanzee loud calls

**DOI:** 10.7717/peerj.5079

**Published:** 2018-06-28

**Authors:** Ammie K. Kalan, Christophe Boesch

**Affiliations:** 1 Department of Primatology, Max Planck Institute for Evolutionary Anthropology, Leipzig, Germany; 2 Wild Chimpanzee Foundation, Leipzig, Germany

**Keywords:** Male signaling, Male competition, Multimodal communication, Leaf clipping, Pant hoot, Tool use, Animal vocalizations, Bioacoustics

## Abstract

Loud calls are used by many species as long-distance signals for group defense, mate attraction, and inter- and intragroup spacing. Chimpanzee loud calls, or pant hoots, are used in a variety of contexts including group coordination and during male contests. Here, we observed an alpha male takeover in wild chimpanzees (*Pan troglodytes verus*) during which the leaf clipping gesture re-emerged after disappearing for almost two years in this community. Leaf clipping only occurred in males and was observed almost exclusively prior to pant hoot vocalizations, as has been observed in other chimpanzee communities of the Taï forest in Côte d’Ivoire. Consequently, we hypothesized that leaf clipping may be important for male-male competition by affecting variation in the acoustic properties of male chimpanzee loud calls. We therefore investigated whether pant hoots preceded by leaf clipping differed acoustically from those without, while also testing the influence of social context on pant hoot variation, namely male dominance rank and hierarchy instability, i.e., before, during and after the alpha takeover. We found that pant hoots preceded by leaf clipping were longer, contained more call elements and drum beats, and lower fundamental and peak frequencies. Moreover, during the alpha takeover pant hoots were shorter, contained fewer drum beats and higher fundamental frequencies. Additionally, pant hoot and aggression rates were also highest during the alpha takeover with leaf clipping more likely to occur on days when pant hooting rates were high. Overall social rank had limited effects on pant hoot variation. We suggest that elevated arousal and aggression during the alpha takeover triggered the re-emergence of leaf clipping and the associated acoustic changes in pant hoots. Further research should focus on the potential mechanisms by which leaf clipping is connected to variation in pant hoots and cross-population comparisons of the behaviour.

## Introduction

Long-distance vocalizations in animals primarily function in group spacing, defense and mate competition (Ryan & Kime, 2003; Delgado, 2006). In mammals, long-distance ‘loud calls’ are central to male displays that are used to deter potential competitors and attract mates, where listeners can obtain information about the dominance or competitive strength of a male signaler (Fischer et al., 2004; Reby et al., 2005; Pitcher et al., 2014; Benítez et al., 2016). Given the source-filter theory for vocal sound production (Fitch & Hauser, 2003), larger males are expected to produce lower pitched calls which can serve as reliable cues of their body size and thus competitive ability (Davies & Halliday, 1978; Fitch, 1997). Support for this relationship has been observed in a variety of birds (Searcy & Andersson, 1986; Gil & Gahr, 2002; Nolan & Hill, 2004), frogs (Davies & Halliday, 1978; Searcy & Andersson, 1986; McClelland, Wilczynski & Ryan, 1996) and mammals (Fitch, 1997; Reby & McComb, 2003; Sanvito, Galimberti & Miller, 2007; Vannoni & McElligott, 2008; Neumann et al., 2010; Puts et al., 2016). Numerous studies have also demonstrated that males in better physical condition usually produce a larger number of calls, call at higher rates or with a longer duration compared to other males (birds (Searcy & Andersson, 1986; Gil & Gahr, 2002; Nolan & Hill, 2004), frogs (Searcy & Andersson, 1986; Welch, Semlitsch & Gerhardt, 1998), hyenas (East & Hofer, 1991) and deer (Reby et al., 2005; Pitcher et al., 2014)). However, studies on non-human primates (‘primates’ hereafter) have provided mixed results on male traits and vocalization parameters (Mitani, 1985; Clark, 1993; Kitchen et al., 2003; Wich et al., 2003; Ey, Pfefferle & Fischer, 2007; Neumann et al., 2010; Barelli et al., 2013; Puts et al., 2016; Benítez et al., 2016). For example, males having a high dominance rank have been associated with low fundamental frequencies in some species (Neumann et al., 2010; Benítez et al., 2016), and higher fundamental frequencies in others (Fischer et al., 2004; Barelli et al., 2013).

The chimpanzee loud call, the ‘pant hoot’, is acoustically sexually dimorphic (Marler & Hobbett, 1975; Clark, 1993; Puts et al., 2016), as well as being individual (Mitani & Brandt, 1994; Kojima, Izumi & Ceugniet, 2003; Notman & Rendall, 2005) and group specific (Crockford et al., 2004). The pant hoot functions in coordinating group movement (Mitani & Nishida, 1993; Fedurek, Donnellan & Slocombe, 2014) and territory defense (Wilson & Wrangham, 2003), while little is known about its role in regulating within group male-male competition (Muller & Mitani, 2005). The pant hoot is a compound call traditionally described as consisting of four phases: introduction, build-up, climax and let-down (Marler & Hobbett, 1975; Crockford et al., 2004; Notman & Rendall, 2005). Male chimpanzees often incorporate buttress drumming into the climax phase of their pant hoot, where the soles of the hands and feet are hit repeatedly against buttress roots of trees (Arcadi, Robert & Boesch, 1998). The highest ranking male, the alpha, is often the most vocal (Clark, 1993; Fedurek et al., 2016), as in other mammals (Pitcher et al., 2014), but little is known about the variation in acoustic properties of the pant hoot beyond individual differences (Marler & Hobbett, 1975; Mitani & Brandt, 1994; Kojima, Izumi & Ceugniet, 2003; Notman & Rendall, 2005). A recent study showed that pant hoot rates of male chimpanzees were positively correlated with urinary testosterone levels and males with higher testosterone produced higher peak frequencies in the climax phase (Fedurek et al., 2016).

In addition to buttress drumming, chimpanzees produce other gestural signals in combination with pant hoots. In the Taï forest in Côte d’Ivoire, males occasionally leaf clip immediately preceding their loud call vocalizations (Boesch, 1995). Leaf clipping is a tool-use gesture where a chimpanzee detaches leaves and rips the leaf blade repeatedly between pressed lips or teeth without ingesting it, often producing an audible ‘ripping’ sound (Nishida, 1980; Boesch, 1995). It has been documented in multiple chimpanzee populations where it appears to be used in different contexts (Nishida, 1980; Sugiyama, 1981; Boesch, 1995; Watts, 2007) and is therefore considered to be one of many cultural variants present in this species (Whiten et al., 1999; Boesch, 2012). The combination of the leaf clip gesture with the pant hoot vocalization is an example of multisensory communication in chimpanzees, also referred to as a ‘free’ multimodal signal (Higham & Hebets, 2013; Wilke et al., 2017), albeit these two signals have limited temporal overlap. Moreover, both signals on their own are also multimodal: comprised of simultaneous audible, facial and gestural components (Liebal et al., 2013; Video S1). The majority of chimpanzee, and other nonhuman primate vocalizations, are actually ‘fixed’ multimodal signals, due to the simultaneous coupling of facial expressions with vocalizations that are necessary for producing particular sounds (Higham & Hebets, 2013; Wilke et al., 2017). Despite the variable definition of the term ‘multimodal’ in animal communication research (Hobaiter, Byrne & Zuberbühler, 2017), there is no doubt that a multimodal framework can demonstrate subtle signaling complexities that can otherwise go unnoticed when restricting analyses to a single modality (Liebal et al., 2013; Wilke et al., 2017). Therefore, we specifically investigated the effect on chimpanzee pant hoots when coupled with leaf clipping because the combination of these signals suggests a degree of flexibility that had hitherto not been examined.

Here, we documented the re-emergence of the leaf clipping gesture during an alpha male takeover in a habituated chimpanzee community (Boesch & Boesch-Achermann, 2000). Almost two years had passed since the last time leaf clipping had been observed in this group. At Taï, leaf clipping is primarily produced by adult males in contexts of social frustration where it is done immediately preceding a pant hoot vocalization and is rarely produced on its own (Boesch, 1995). To our knowledge, leaf clipping occurs in both sexes in other chimpanzee populations and appears to be disassociated from the pant hoot vocalization altogether (Boesch, 2012). For example, in Mahale, males and estrus females use leaf clipping to initiate copulations (Nishida, 1980), and this is similarly observed in Budongo (Hobaiter & Byrne, 2014), Gombe and Ngogo (Watts, 2007). Meanwhile, male and female chimpanzees in Bossou, Guinea have been observed to engage in leaf clipping in a variety of contexts including frustration, copulation and play (Sugiyama, 1981). However, due to its relatively low rate of occurrence in wild populations (Boesch, 1995; Watts, 2007) and the lack of information about this nuanced behaviour, it remains a poorly understood socio-cultural trait in wild chimpanzees.

The aim of this study was therefore to assess acoustic variation in the pant hoot with respect to the occurrence of leaf clipping while also investigating the effects of male dominance rank and male-male competition during the alpha takeover period on chimpanzee loud calls. We specifically investigated acoustic cues typically associated with male competitive ability in the vocalizations of primates and other mammals (Clark, 1993; Wich et al., 2003; Reby et al., 2005; Neumann et al., 2010; Pitcher et al., 2014; Benítez et al., 2016), and predicted that leaf clipping and higher ranking males would produce pant hoots with lower fundamental frequencies, a longer duration, and contain more call elements and buttress drumming. Additionally, since disruptions in the dominance hierarchy are expected to increase male-male competition (Muller & Mitani, 2005; Georgiev, 2012), we predicted that the alpha male takeover provided a critical social context for male signals to be modulated, including pant hoots and leaf clipping at Taï. Therefore, we further tested whether daily rates of male pant hooting and aggression rates were also affected by the period of instability (i.e., before, during and after the alpha takeover) and the occurrence of leaf clipping.

## Materials & Methods

All data were collected between July 2011 and May 2012 in the Taï National Park in Côte d’Ivoire. The study subjects were five males from one chimpanzee community, the South group (Boesch, 2012), including three adult males (16, 18 and 18 years of age) and two sub-adult males both 13 years of age. All day focal follows were conducted on the five males with the help of a field assistant for a total of 666 h of observation during which all behavioural activities, social interactions, and vocalizations were continuously noted (Table 1). There were a total of 68 focal follow days for the five males (mean: 9.79 h; range: 3–12.5 h). The sub-adult males were only followed once it became clear they attained top rank positions (3rd and 4th rank) in the hierarchy due to the small group size (19 adults and sub-adult individuals plus 5 infants). All data were collected on wild chimpanzees using non-invasive, observational methods only.

**Table 1:** Summary of the focal follow data for each male chimpanzee. The total number of focal observation hours per individual before, during and after the alpha takeover and the total number of pant hoots and aggressive interactions (focal could be aggressor or victim) observed during these focal follows.

	Hours of observation from focal follows			# of pant hoots emitted	# of aggressive interactions
	Before	During	After		
Jacobo	NA	11.5	45	75	21
Kuba	89.5	27.5	96.5	235	56
Romario	NA	NA	74	121	8
Utan	48.5	11.5	23.5	64	34
Woodstock	99	25.5	114	148	38

In the field, we noted whether a pant hoot was directly preceded by leaf clipping (<3s before the start of a pant hoot). Soft, intermittent ‘hoos’ could sometimes be heard whilst the individual leaf clipped (Video S1), however only if these ‘hoos’ graded into the start of a pant hoot (<3s) were they considered as part of the pant hoot. Leaf clipping observations and recordings of pant hoots (see Acoustic Analyses) were collected during focal follows and *ad libitum* throughout the study period whenever target males were present (Table 2). Dominance ranks were observed to change twice during the study period: (1) due to the alpha male takeover where the beta and alpha male switched ranks, and (2) due to the disappearance of a high ranked male (Utan) whereby absolute ranks of all individuals changed. Male ranks were assigned by continuous observations of the pant grunt vocalization, specifically the directionality of pant grunts among males since this vocalization is uniquely produced up the hierarchy as an overt signal of submission towards individuals of a higher rank (Boesch & Boesch-Achermann, 2000; Muller & Mitani, 2005). For all pant hoots in this study, we assigned the dominance rank of the male caller based on their rank at the moment the call was emitted.

**Table 2:** Names and rank(s) held during the eleven month study period for each male chimpanzee. The number of high-quality pant hoot recordings collected before, during and after the alpha takeover that were used for acoustic analyses. Of these, the number of pant hoot recordings immediately preceded by a leaf clip per individual.

	Rank(s)^a^	# of pant hoots recorded for analyses			# of recorded pant hoots preceded by leaf clipping		
		Before	During	After	Before	During	After
Jacobo	4, 3	0	3	19	0	0	0
Kuba	2, 1	9	37	46	0	8	6
Romario	5, 4	0	2	46	0	0	9
Utan	3	10	9	2	0	0	0
Woodstock	1, 2	10	9	10	0	1	3

**Notes.**

a Ranks males held throughout the study period, 1  = alpha.

The study duration was divided into three periods: ‘before’ (three months), ‘during’ (one month) and ‘after’ (six months) the alpha male takeover to describe the relative instability in the male hierarchy based on critical observations of physical aggression (first fight observed between the alpha and beta male on October 16, 2011) and the alpha male finally conceding to the beta male by clearly pant grunting to him on November 19, 2011. Therefore, the duration of each period had to be deduced *post hoc* for these analyses according to the behaviour of the males. Permissions for field research were granted by the Ministère de la Recherche Scientifique, the Ministère de l’Environnement et des Eaux et Forêts and the Office Ivorien des Parcs et Reserves of Côte d’Ivoire (Ref: 11/MINEF/OIPR/DT/CAT).

### Acoustic analyses

Recordings of pant hoot vocalizations were made by AK using a Marantz PMD661 solid state recorder and a Sennheiser ME66/K6 directional microphone handheld with a windshield using a 44 kHz sampling frequency at 24 bits/s. Only recordings where the caller’s identity was certain were used and the pant hoot had to be free of any other individual’s vocalizations. All pant hoots were recorded at a distance of 3 to 10m from the vocalizing chimpanzee. Pant hoots were recorded whenever possible throughout the study period (Table 2) and our final dataset consisted of pant hoots emitted while male chimpanzees were resting (*n* = 46), traveling (*n* = 124), feeding, or arriving to a feeding tree (*n* = 42). For our analyses we did not distinguish between each of these behavioural activities due to the small sample size per individual per category which caused model instability (but see *Statistical Analyses* and Table S1). In the field it was noted whether any portion of the call was missing from the recording (incomplete) or whether the pant hoot was recorded in its entirety (complete). Incomplete recordings occurred for 88 of the 212 pant hoots used in these analyses for multiple reasons: noisy recording due to microphone or cable damage, caller moving quickly while vocalizing, background noise or other chimpanzees calling, but in all cases at least one of the three phases was recorded in its entirety. Chimpanzees at Taï rarely include a let-down phase (Arcadi, Robert & Boesch, 1998); therefore, it was not included in our analyses because it was not observed.

**Figure 1:**
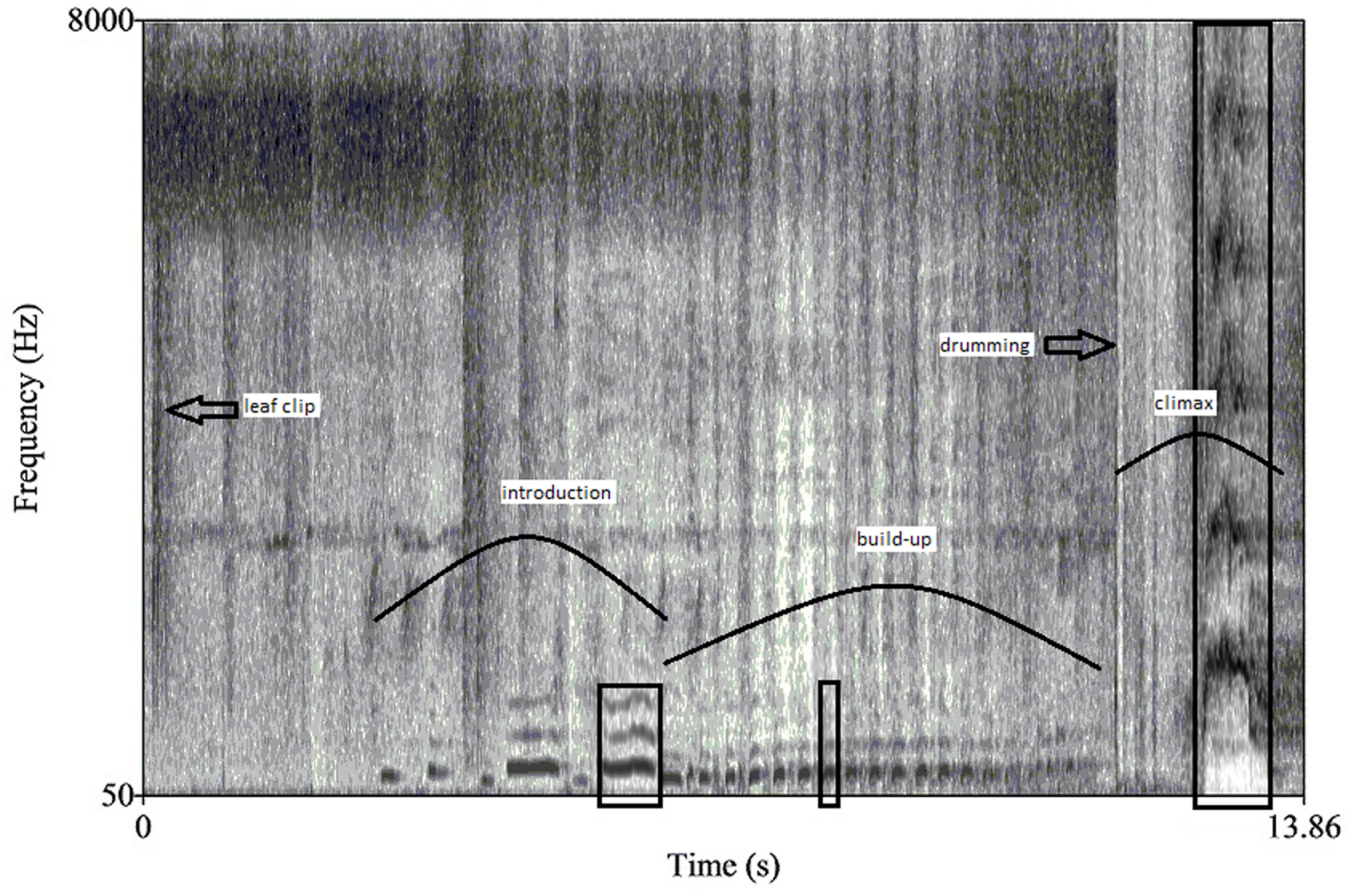
A spectrogram of a male chimpanzee pant hoot vocalization preceded by leaf clipping. Curved lines identify the three main phases: introduction, build-up and climax while boxes denote the call targeted for further analyses within each phase. Both leaf clipping and buttress drumming also occur in this pant hoot and are indicated with arrows.

All pant hoot measurements were conducted using the speech analysis freeware Praat version 5.3 (Boersma, 2001). We calculated acoustic parameters that have already been shown to vary with male quality or dominance in other mammals, including chimpanzees, namely phase and call durations, peak and fundamental frequencies of specific calls, and number of call units per phase of the pant hoot (Marler & Hobbett, 1975; Clark, 1993; Mitani, Hunley & Murdoch, 1999; Fedurek et al., 2016). Acoustic measurements were done via visual inspection of a spectrogram whilst simultaneously listening to the pant hoot. Spectrogram settings were always set to a 50 to 8,000 kHz viewing range using a window length of 0.01s. The pant hoots were visually separated into the introduction, build-up and climax phase that are well described and easily discernible (Marler & Hobbett, 1975; Mitani & Brandt, 1994; Crockford et al., 2004; Notman & Rendall, 2005; Fig. 1). The three remaining phases could include a variable number of inhaled and exhaled call elements with the exception of the climax phase which sometimes had no vocal elements but only buttress drumming. Only the number of voiced call elements and/or drum beats in each phase was counted. Pant hoots without climax screams are often produced by chimpanzees, particularly females (Marler & Hobbett, 1975) and males in this study produced pant hoots which ended without a climax scream and only buttress drumming (57/212 pant hoots in this study). Additionally, males at Taï buttress drum more than other populations and generally do so in conjunction with pant hoots (Arcadi, Robert & Boesch, 1998); therefore, we felt it was important to consider drumming as an integral part of the pant hoot for this population (126/212 pant hoots included drumming).

Durations were measured for the total pant hoot and for each of the three phases as well as the drumming bout, excluding leaf clipping, if it occurred (from the onset of the first voiced call or drum beat to the end of the last call or drum beat). The drumming bout was usually solely confined to the climax phase but sometimes overlapped with the end of the build-up. We also measured duration and frequency parameters from a single call isolated from each of the three phases following methods similar to previous studies of chimpanzee pant hoots (Mitani, Hunley & Murdoch, 1999; Crockford et al., 2004). For example, we selected the middle call of the build-up for analyses since the build-up largely consists of unmodulated calls. If the build-up had an even number of total voiced call elements, we considered the next element as the middle call of the build-up (i.e., for a build-up phase with eight calls we analyzed number five). For the introduction phase we selected the last call for analyses since sometimes the first calls of the pant hoot were missing or of poor recording quality (i.e., incomplete). For the climax, the call with the greatest peak frequency was selected, again as in other studies (Mitani, Hunley & Murdoch, 1999; Fig. 1 for overview of pant hoot variables).

The fundamental frequency (F0) and peak frequency (pF) were measured for selected calls using the spectral slice tool which automatically calculates a power spectrum of a selected call. The first peak in the spectral slice corresponded to the F0 and the peak with the highest relative amplitude the pF. Values for fundamental and peak frequencies were also verified visually. In total we had 18 quantitative variables assessed for 212 pant hoots produced by 5 chimpanzee males.

**Table 3:** Summary of the 18 GLMMs testing for various acoustic parameters of pant hoots produced by five chimpanzee males. Test predictors included period of instability, caller rank, and whether leaf clipping preceded the pant hoot. Sample size refers to the total number of pant hoots per GLMM.

	Response variable	Transformation of response	Error structure (link function)	Sample size	Full vs null model comparison
	Total duration (s)	sqrt	Gaussian (identity)	212	*χ*^2^ = 15.03, *df* = 4, *P* = 0.0046
Introduction	# calls in the introduction	none	Poisson (log)	173	*χ*^2^ = 12.56, *df* = 4, *P* = 0.014
	Introduction duration (s)	sqrt	Gaussian (identity)	173	*χ*^2^ = 12.52, *df* = 4, *P* = 0.014
	F0 of last call of the introduction (Hz)	sqrt	Gaussian (identity)	173	*χ*^2^ = 5.67, *df* = 4, *P* = 0.23
	Duration of the last call of the introduction (s)	none	Gaussian (identity)	173	*χ*^2^ = 5.35, *df* = 4, *P* = 0.25
	pF of the last call of the introduction (Hz)	log	Gaussian (identity)	173	*χ*^2^ = 6.10, *df* = 4, *P* = 0.19
Build-up	# of voiced calls in the build-up	none	Negative Binomial (log)	189	*χ*^2^ = 12.16, *df* = 4, *P* = 0.016
	Duration of the build-up (s)	sqrt	Gaussian (identity)	189	*χ*^2^ = 0.00, *df* = 4, *P* = 1.00
	F0 of the middle call of the build-up (Hz)	sqrt	Gaussian (identity)	189	*χ*^2^ = 18.10, *df* = 4, *P* = 0.0012
	Duration of the middle call of the build-up	log (×1000)	Gaussian (identity)	189	*χ*^2^ = 13.87, *df* = 4, *P* = 0.0077
	pF of the middle call of the build-up (Hz)	log	Gaussian (identity)	189	*χ*^2^ = 16.11, *df* = 4, *P* = 0.0029
Climax	# of elements in the climax	none	Negative Binomial (log)	189	*χ*^2^ = 28.59, *df* = 4, *P* = 0.00001
	Duration of the climax (s)	sqrt	Gaussian (identity)	189	*χ*^2^ = 21.61, *df* = 4, *P* = 0.00024
	F0 of the highest call of the climax (Hz)	none	Gaussian (identity)	127	*χ*^2^ = 7.27, *df* = 4, *P* = 0.12
	Duration of the highest call of the climax (s)	none	Gaussian (identity)	127	*χ*^2^ = 2.46, *df* = 4, *P* = 0.65
	pF of the highest call of the climax (Hz)	log	Gaussian (identity)	127	*χ*^2^ = 11.28, *df* = 4, *P* = 0.024
	Duration of drumming (s)^a^	sqrt	Gaussian (identity)	210	*χ*^2^ = 33.06, *df* = 4, *P* < 0.00001
	# of drum beats^a^	none	Negative Binomial (log)	210	*χ*^2^ = 24.01, *df* = 4, *P* = 0.00008

**Notes.**

a Drumming could start in the build-up or climax but usually occurred solely in the climax phase.

### Statistical analyses

All statistical analyses were conducted in R version 3.2.4 (R Core Team, 2017). We fitted linear mixed models (LMM) and Generalized Linear Mixed Models (Baayen, 2008; Bates et al., 2015) to test the effects of male rank, period of male instability, and leaf clipping on the 18 acoustic variables measured from a total of 212 pant hoots produced by five chimpanzee males of a single community, the South group. Not all pant hoots contained all applicable variables measured therefore sample size varied among models (Table 3). Importantly, we opted to test each acoustic variable separately rather than conduct factor analyses because with a long, compound call such as a pant hoot, one phase of the vocalization does not necessarily constrain other phases, and because we wanted to be able to compare our results with previous chimpanzee studies which investigated particular parts of the pant hoot. Since the number of males in the group differed throughout the study period, before fitting models the value for male rank was standardized to range from 0 to 1. All models were fitted using the functions ‘lmer’,’glmer’ or ’glmer.nb’ of the package lme4 in R (Bates et al., 2016). The response variables were the acoustic variables, and the fixed effects always included the three test predictors (period (before, during, after), leaf clipping (Y/N), and rank) and one control predictor of whether the recording included the complete pant hoot produced by the chimpanzee or not (Y/N). All models also included the random effect for caller ID and the random slopes of all fixed effects within caller ID as centered dummy variables (Schielzeth & Forstmeier, 2009; Barr et al., 2013). We included behavioural activity, (traveling, feeding and resting) for each pant hoot as an additional control variable but the low sample size per individual in the three contexts led to model instability. Therefore, once we ensured that model results did not change with behavioural activity included as a control (Table S1) we were able to confidently exclude it from final models to get reliable estimates and variances for the test predictors of interest.

All continuous response variables (durations, F0 and pF variables) were analysed using LMMs (i.e., with a Gaussian error structure and identity link function) with the argument REML set to false in order to assess model significance using likelihood ratio tests. The single Poisson model for the number of calls in the introduction was fitted using the function ‘glmer’ of the package lme4 with the argument family set to Poisson and using a log link function (Bates et al., 2016). We fitted negative binomial models for three response variables using the function ‘glmer.nb’ with a log link function (Bates et al., 2016): number of voiced elements in the build-up, the number of elements in the climax and number of drum beats. None of the Poisson and negative binomial models suffered from overdispersion (all disperison parameters < 1.13; Dobson & Barnett, 2008). Gaussian models were checked for normally distributed and homogeneity of residuals by visual inspections of QQ-plots and residuals plotted against fitted values which did not indicate any violation of these assumptions. Additionally, all models were assessed for stability by verifying that model estimates did not vary greatly when individuals were removed one at a time. We further checked for collinearity among predictors by determining Variance Inflation Factors (VIF; Bowerman & O’Connell, 2000) using the function ‘vif’ of the package car on a linear model with no random effects included (Fox & Weisberg, 2001). All VIFs were between 1.01–1.06 and therefore were no cause for concern. Model significance was assessed using a likelihood ratio test comparing the full versus null model using the function ‘anova’ with a Chisq approximation (Forstmeier & Schielzeth, 2011). The null model lacked the fixed effects of period, rank and leaf clipping but was otherwise identical to the full model. If this was significant (*P* < 0.05) we went on to assess the significance of the individual test predictors using a likelihood ratio test with the help of the ‘drop1’ function in R set to using a Chisq approximation (Dobson & Barnett, 2008; Barr et al., 2013).

Since we fitted a total of 18 models, one for each of the acoustic variables determined from the same set of calls, the tests were not independent and therefore required a correction for multiple testing. We used the procedure proposed by Potter and Griffiths (Potter & Griffiths, 2006) which is a modification of Fisher’s Omnibus test (Haccou & Meelis, 1994) accounting for non-independence of the tests by deploying a permutation procedure (Adams & Anthony, 1996; Manly, 1997). In brief, this approach consists of repeatedly randomizing (‘permuting’) all response variables simultaneously (i.e., the correlations among the call parameters are retained) and then fitting the respective model for each of the permuted data sets. To further account for the non-independence of calls recorded from the same individuals we restricted the randomizations to take place only within individuals. We conducted 1,000 permutations into which we included the original data as one permutation. For each of the permuted data sets we fitted the same models as for the original data and conducted a full null model comparison for each of the 18 acoustic parameters as described above. We then combined the derived *P*-values into a single test statistic using *ts* =  − 2 × Σlog_*e*_(*p*) to obtain the Chi-squared distribution, as expected under the null hypothesis and accounting for the independence of the *P*-values, from the 1,000 full null model comparison *P*-values. Finally, we determined the overall *P*-value as the proportion of permutations revealing a test statistic at least as large as that obtained from the original data set. This revealed an overall *P* value (accounting for multiple correlated tests) of 0.002 meaning that our test predictors significantly explained variation in the acoustic parameters.

We fitted two additional GLMMs to assess the effect of period, rank and leaf clipping on rates of male pant hoot production and aggression based solely on focal follow data of the males (Table 1). Both GLMMs had a negative binomial error structure with a log link function and were fitted using the function ‘glmer.nb’ of the package lme4 (Bates et al., 2016) with the total number of pant hoots or total number of aggressive interactions as the response variables, respectively. Pant hoots included calls with or without buttress drumming components, and aggressive interactions included all male displays (directed or undirected at conspecifics), chasing and hitting (Muller & Mitani, 2005), irrespective of whether the focal male was the aggressor or the victim in these interactions. The fixed effects included the three predictors: period, rank and leaf clipping as in the previous models. We also included an offset term for the number of hours (log transformed; McCullagh & Nelder, 1989) the individual was followed during a given day to control for observation effort (Dobson & Barnett, 2008). For the aggression rates model, rank was kept as a control fixed effect since we were not particularly interested in rank related effects on aggression and expected higher ranking individuals to be more aggressive, given the way in which chimpanzee dominance is exerted and maintained (Boesch & Boesch-Achermann, 2000; Muller & Mitani, 2005). A random effect for focal ID was included, along with all random slopes for the fixed effects within focal ID (Barr et al., 2013). Again, VIFs and dispersion parameters were calculated as explained above and indicated no issues (maximum dispersion parameter: 1.11; maximum VIF 1.37). Additionally, model stability was evaluated as described above, and significance of both models was assessed using a likelihood ratio test in comparison with a respective null model which consisted of only the offset, control predictor (if applicable) as well as random effects and random slopes (Forstmeier & Schielzeth, 2011).

## Results

At the time of the study, the chimpanzees of this community had not been seen to leaf clip since December 2009 when the group had lost half of its members during a respiratory disease outbreak (unpublished data). None of the remaining adult males were observed to leaf clip following this outbreak despite frequent observations of leaf clipping in the neighbouring habituated community (AK Kalan, pers. obs., 2010–2011). We then observed the first alpha male takeover in this group following the outbreak. A physical fight was observed on the evening of October 16, 2011 between the alpha and beta male. The beta male at the time, Kuba, was the first individual observed to leaf clip on October 17, 2011 and all other males of the community were present at the time. On October 23, 2011 the contested alpha male, Woodstock, also began to leaf clip and only the beta and alpha male were seen to leaf clip until January 18, 2012 when the 5th ranked male at the time, Romario, began to leaf clip. Around this same time, the 3rd ranked male, Utan, disappeared whose body was never recovered. After their first occurrences of leaf clipping all three males continued to occasionally leaf clip but no other male in the group was seen to start leaf clipping during the study period. The leaf clip gesture primarily occurred preceding a pant hoot vocalization, and was thus produced sequentially, not simultaneously (Video S1). We observed a total of 36 leaf clips by the three males during the 11 month study period, 33 of which directly preceded a pant hoot vocalization and of these, 27 pant hoots were recorded and therefore were part of these analyses (Table 2). The remaining three leaf clip observations occurred after a pant hoot had ended or accompanied a directed charge at a conspecific without a pant hoot. Of all 36 leaf clipping observations, 10 were accompanied by a direct charge at a conspecific, in 11 cases a female chimpanzee in estrus was present, and in five cases the caller was alone with no other conspecific in sight whilst leaf clipping. Therefore, an audience was visually present for 86% (31/36) of the leaf clipping observations.

Of the 18 acoustic parameters tested in GLMMs for the influence of leaf clipping, period of instability, and rank, 12 had significant full versus null model comparisons (all *P* < 0.05; Table 3). Of these 12 acoustic variables, five measured durations (s) of the whole loud call or parts of the pant hoot, three were the number of call and/or drum elements in the introduction, build-up, and climax phase, and one was the total number of drum beats. The other three significant acoustic parameters were related to frequency parameters; namely, the fundamental frequency (F0) and peak frequency (pF) of the middle call of the build-up and the maximum pF recorded in the climax (Table 3). For eight of the 12 models, the control variable complete or incomplete pant hoot recording had a significant impact in these models, controlling for the fact that incomplete recordings were more likely to be shorter or have fewer elements as expected. Therefore, all of our results controlled for the bias possible with an incomplete recording.

Only three acoustic parameters of the pant hoot were affected by the rank of the male chimpanzee: the introduction phase was shorter (est. ± SE: −0.39 ± 0.10, *χ*^2^ = 6.90, *df* = 1, *N* = 173, *P* = 0.0086; Fig. S1) and contained fewer call elements for higher ranking males (−0.58 ± 0.11, *χ*^2^ = 9.31, *df* = 1, *N* = 173, *P* = 0.0023; Fig. S1) while the number of voiced calls in the build-up phase was greater for higher ranking males (0.55 ± 0.21, *χ*^2^ = 8.07, *df* = 1, *N* = 189, *P* = 0.0045; Table 4).

**Table 4:** Summary of the direction of significant effects (*P* < 0.05) of the three predictors on the twelve acoustic pant hoot variables which revealed significant full versus null model comparisons.

	Period of instability	Leaf clipping	Rank
Total duration	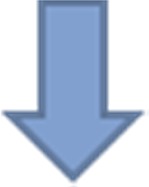	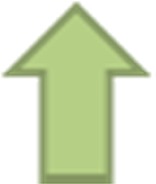	_
# of calls in introduction phase	_	_	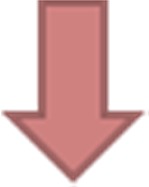
Duration of introduction phase	_	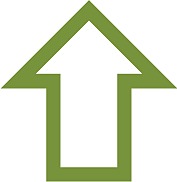	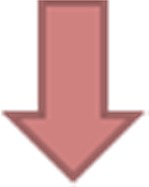
# of voiced calls in build-up phase	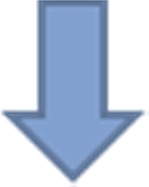	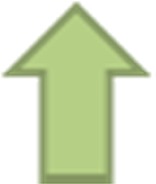	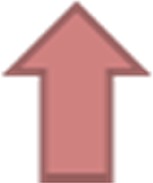
F0 of the middle call of build-up phase	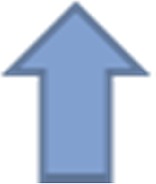	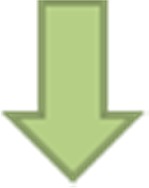	_
Duration of the middle call of the build-up	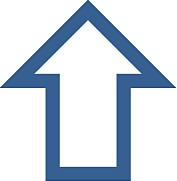	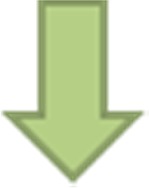	_
pF of the middle call of build-up phase	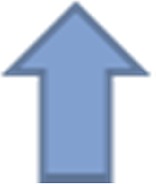	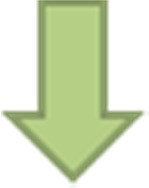	_
# of elements in climax phase	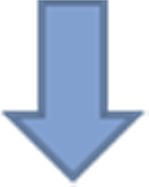	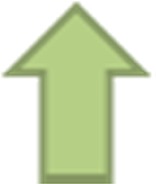	_
Duration of climax phase	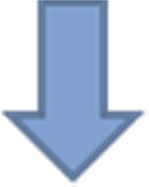	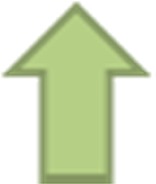	_
The maximum pF of a climax call	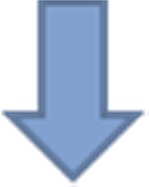	_	_
Duration of total drumming	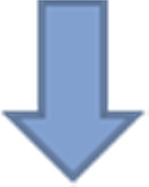	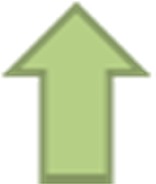	_
# of drum beats	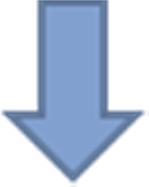	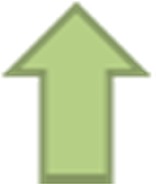	_

**Notes.**

(Period of Instability: during and/or after the alpha takeover occurred relative to before; Leaf clipping: when leaf clipping preceded the pant hoot; Rank: as rank increases in dominance; unfilled arrows *P* < 0.1).

**Figure 2:**
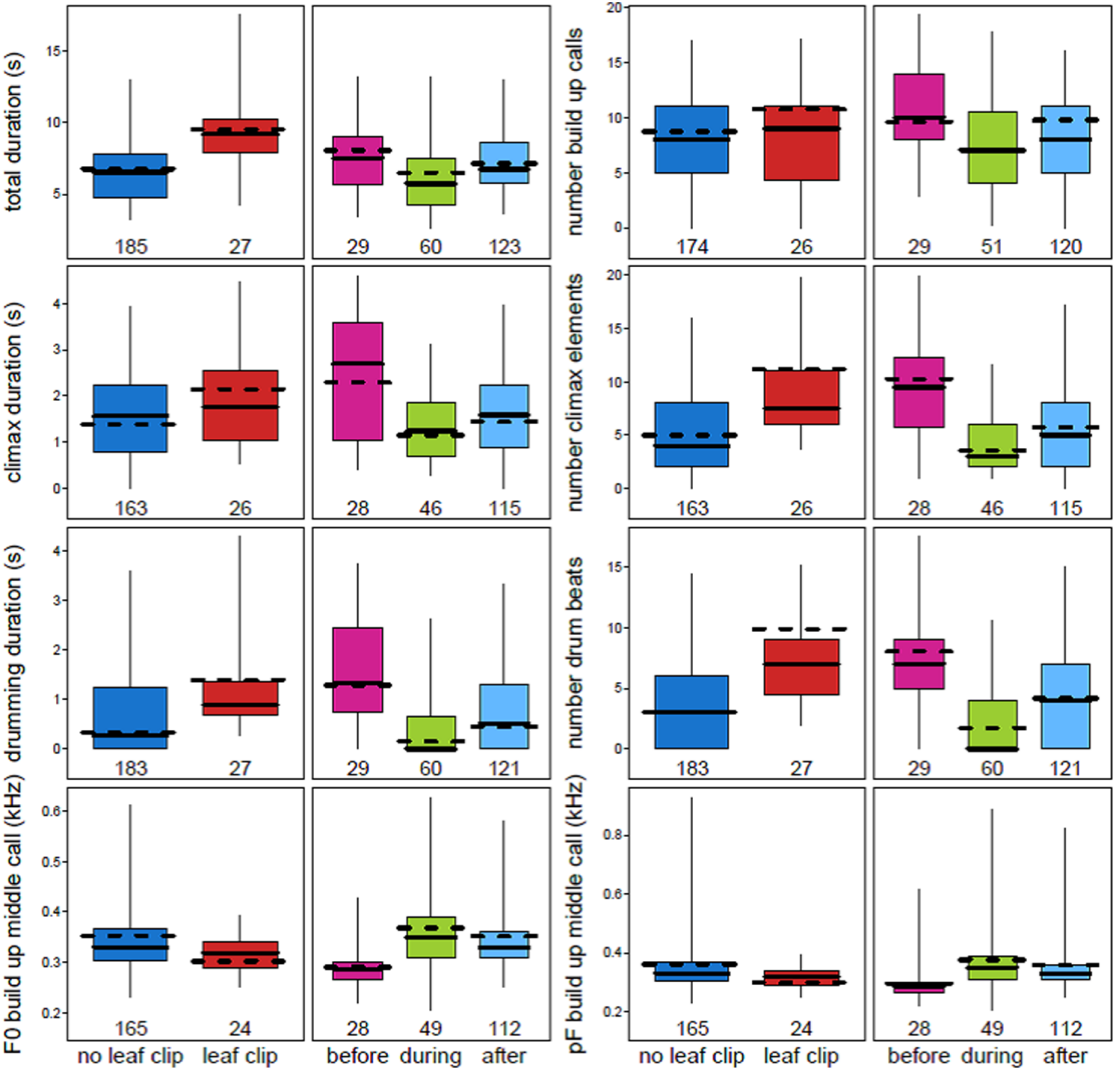
Variation in eight acoustic parameters of male chimpanzee pant hoots that were significantly affected by both leaf clipping and the period of instability. Plots show the median (solid horizontal line) for each acoustic parameter. The boxes represent quartiles and the vertical lines show percentiles (2.5 and 97.5%). The *y*-axis is the acoustic parameter and the *x*-axis shows the levels of the two factors: leaf clipping and period of instability (before, during and after the alpha takeover). The dashed horizontal line shows the model prediction given all other fixed effects being at their average value.

Multiple acoustic parameters of the loud call were found to change when a male chimpanzee leaf clipped immediately before emitting a pant hoot (Fig. 2; Table 3; Table S2). Leaf clipping was associated with longer pant hoots overall (est. ± SE: 0.48 ± 0.09, *χ*^2^ = 9.23, *df* = 1, *N* = 212, *P* = 0.0024). Following leaf clipping the durations of the introduction (0.42 ± 0.11, *χ*^2^ = 3.74, *df* = 1, *N* = 173, *P* = 0.053) and climax phases (0.29 ± 0.09, *χ*^2^ = 5.04, *df* = 1, *N* = 189, *P* = 0.025) were longer although the duration of the middle call of the build-up was shorter (−0.34 ± 0.09, *χ*^2^ = 7.74, *df* = 1, *N* = 189, *P* = 0.0054). Additionally, when leaf clipping occurred there were more call elements in the build-up (0.21 ± 0.12, *χ*^2^ = 4.33, *df* = 1, *N* = 189, *P* = 0.037) and climax (0.81 ± 0.15, *χ*^2^ = 7.89, *df* = 1, *N* = 189, *P* = 0.0051). With respect to buttress drumming, the total duration of drumming was longer when leaf clipping occurred (0.60 ± 0.12, *χ*^2^ = 8.84, *df* = 1, *N* = 210, *P* = 0.0029) and there were also more drum beats produced by the caller (1.17 ± 0.28, *χ*^2^ = 7.62, *df* = 1, *N* = 210, *P* = 0.0058; Fig. 2; Tables 3 and 4; Table S2).

Many of the effects observed for leaf clipping on the acoustic properties of pant hoots differed with respect to the effects of instability period on those same acoustic parameters (Fig. 2; Tables 3 and 4). In particular, the duration of the total pant hoot was shorter during and after the alpha takeover (*χ*^2^ = 6.28, *df* = 2, *P* = 0.043; *during*: est. ± SE: −0.27 ± 0.12; *after*: −0.21 ± 0.09); the number of voiced calls in the build-up was also lower during the period of instability (*χ*^2^ = 6.42, *df* = 2, *P* = 0.040; *during*: −0.31 ± 0.12; *after*: −0.09 ± 0.12; Fig. 2) while the duration of the middle call of the build-up phase tended to be longer (*χ*^2^ = 5.19, *df* = 2, *P* = 0.075; *during*: 0.46 ± 0.17; *after*: 0.09 ± 0.08). The F0 of the middle call of the build-up was higher during the alpha takeover (*χ*^2^ = 12.24, *df* = 2, *P* = 0.0022; *during*: 2.21 ±0.67; *after*: 1.61 ± 0.48) and likewise with the pF of the middle call of the build-up (*χ*^2^ = 10.37, *df* = 2, *P* = 0.0056; *during*: 0.26 ± 0.10; *after*: 0.20 ± 0.06; Fig. 2; Tables 3 and 4; Table S2).

With respect to the climax phase of the pant hoot, the duration of the climax was shorter during and after the alpha takeover relative to before it occurred (*χ*^2^ = 16.72, *df* = 2, *P* = 0.00023; *during*: −0.44 ± 0.10; *after*: −0.31 ± 0.09), and drumming duration was also shorter (*χ*^2^ = 24.29, *df* = 2, *P* = 0.000053; *during*: −0.75 ± 0.13; *after*: −0.46 ± 0.12). There were also fewer call elements associated with the climax during the alpha takeover (*χ*^2^ = 20.37, *df* = 2, *P* = 0.000038; *during*: −1.05 ± 0.17; *after*: -0.57 ± 0.15) and fewer drum beats (*χ*^2^ = 17.38, *df* = 2, *P* = 0.00017, *during*: −1.54 ± 0.31; *after*: −0.66 ± 0.28). One additional variable of the climax was also influenced by the period of instability which was the pF of the call with the highest energy in the climax phase (*χ*^2^ = 11.05, *df* = 2, *P* = 0.0040; during: −0.34 ± 0.13; after: −0.53 ± 0.13) where the pF was lower relative to before the dominance hierarchy was disrupted (Fig. 2; Tables 3 and 4; Table S2).

**Figure 3:**
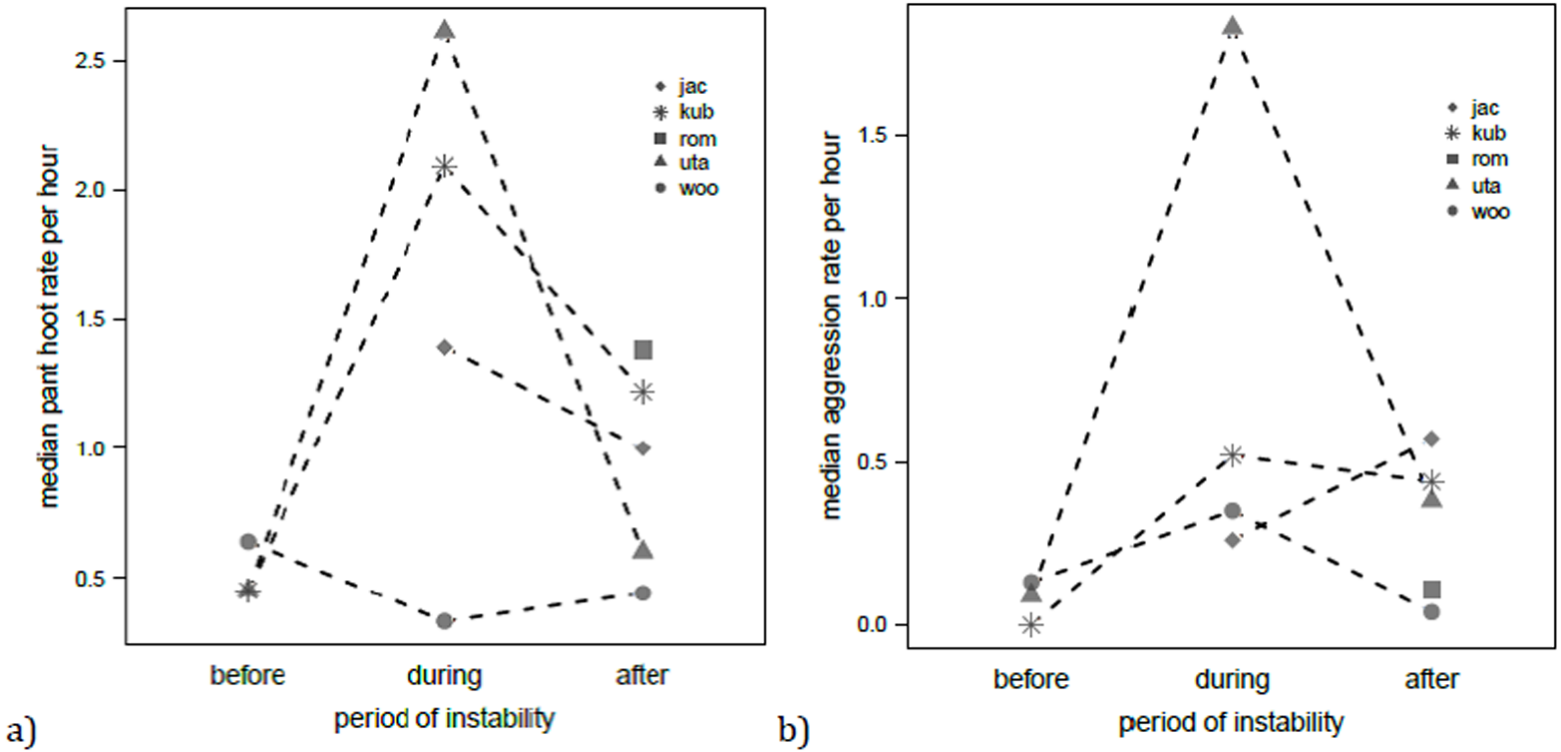
Median rates of pant hooting (A) and aggression (B) per hour for each focal chimpanzee male before, during and after the alpha takeover. Lines connect points of the same respective individual where applicable.

With respect to the rate of daily pant hoot production by males, this was significantly affected by the predictors (full versus null model: *χ*^2^ = 14.24, *df* = 4, *P* < 0.01, *N* = 68). In particular, individual pant hoot rates were highest during the alpha takeover (*χ*^2^ = 6.29, *df* = 2, *P* = 0.043; Fig. 3), and also on days when the focal male was seen to leaf clip (est. ± SE = 0.46 ±0.19, *χ*^2^ = 5.18, *df* = 1, *P* = 0.023). Rank did not significantly influence pant hoot rates (*χ*^2^ = 1.61, *df* = 1, *P* = 0.20). Individual aggression rates were also affected by the predictors (full vs. null model: *χ*^2^ = 10.14, *df* = 3, *P* = 0.017, *N* = 68) where they were elevated during and after the alpha male takeover relative to before (*χ*^2^ = 9.98, *df* = 2, *P* < 0.01; Fig. 3) but leaf clipping had no significant effect on aggression rates (est. ± SE = − 0.07 ± 0.31, *χ*^2^ = 0.053, *df* = 1, *P* = 0.82).

## Discussion

We found strong support for pant hoots associated with leaf clipping being modified in both spectral and temporal acoustic properties. Pant hoots preceded by leaf clipping had longer phases and call durations, contained a greater number of call elements and drum beats, and had lower fundamental and peak frequencies in the build-up phase. We also found that increased dominance hierarchy instability, resulting from greater male-male competition, affected the acoustic properties of male pant hoots. Pant hoots produced by males during and after an alpha male takeover had shorter durations, fewer calls and drum beats, and higher fundamental and peak frequencies in the build-up phase relative to before. Additionally, we found daily male pant hoot rates to be higher when the male hierarchy was unstable and on days when males leaf clipped. Therefore, in line with our predictions, the re-emergence of leaf clipping in this chimpanzee community and increased male-male competition were associated with measurable acoustic variation in male loud calls. Male rank did not influence pant hoot rates and we were generally surprised to find only three acoustic parameters varied according to male rank (Table 4), albeit we only had five males in this community. However, for the majority of our study period the male dominance hierarchy remained precarious due to the 3rd ranked male disappearing soon after the alpha male takeover; consequently, rank may have had a relatively inconsequential effect in our dataset.

With regards to the motivation for leaf clipping, it is likely not a coincidence that the behaviour re-appeared in this community during a time of intense male-male competition. Although leaf clipping was not directly correlated with daily rates of male aggression, the association may be one of a more general context of social frustration as has been described for leaf clipping in other chimpanzee communities (Sugiyama, 1981; Boesch, 1995). Leaf clipping itself is a conspicuous tool-use gesture to receivers in close proximity (Watts, 2007) which may signal a threat of aggression to nearby conspecifics (Boesch, 1995). Indeed, when males begin to leaf clip, nearby conspecifics were often observed to move away from the signaler as if giving him space (AK Kalan, pers. obs., 2011–2012). Although not all males in this study were observed to leaf clip (Table 2) those that did were often observed to be piloerect and swaying back and forth while leaf clipping, clear indications of high arousal (Muller & Mitani, 2005; Clutton-Brock, 2016). In mammals, individuals in an elevated state of arousal often call at higher rates and produce longer calls with higher peak frequencies although this is often true in both positive and negative affective contexts (Briefer, 2012). Additionally, according to Morton’s motivation structural rules (Morton, 1977), animals highly aroused and signaling aggressive intent are expected to produce calls with a lower fundamental frequency which is what we observed in this study when males emitted pant hoots preceded by leaf clipping. Therefore, overall our results support an arousal explanation for the re-emergence of leaf clipping and associated changes to the male chimpanzee loud call.

The contrasting direction of the effects of leaf clipping and hierarchy instability on pant hoot parameters (Table 4; Table S2) additionally suggests that leaf clipping might help to alleviate vocal exhaustion caused by an increased pant hoot rate and increased aggression during the alpha takeover period in this study. Vocal exhaustion is characterized by calls becoming shorter with fewer call elements (Fischer et al., 2004) similar to the pant hoots produced by males during the period of instability in this study (Fig. 2; Table 4; Table S2). It has also been reported that chimpanzees are energetically stressed during periods of elevated male-male competition which could further contribute to poorly produced pant hoots at this time (Georgiev, 2012). However, it is difficult to assess what, if any, physiological benefits leaf clipping could have on pant hoots with respect to sound production or respiration therefore detailed knowledge about chimpanzee vocal production and anatomy is needed to elucidate any potential mechanism at work here. Alternatively, leaf clipping may have emerged as a displacement activity by male chimpanzees to potentially alleviate social stress experienced during this period of hierarchy instability (Maestripieri et al., 1992). However, stress displacement behaviours in animals are usually more self-directed, such as scratching or self-grooming (Maestripieri et al., 1992), and this would not explain why leaf clipping was specifically coupled with pant hoots.

Although the exact mechanism remains to be investigated, at the very least the leaf clipping behaviour does lengthen the male loud call display by combining the gesture and pant hoot into a more complex multimodal signal. In previous research, leaf clipping has been described as an attention grabbing gesture (Watts, 2007); therefore, this behaviour could serve to draw the attention of nearby conspecifics to the signaler and the subsequent pant hoot vocalization. Indeed, audible gestures often form key components of the multimodal signals present in the communicative repertoire of chimpanzees, both in the wild (Wilke et al., 2017; Hobaiter, Byrne & Zuberbühler, 2017) and in captivity (Leavens, Russell & Hopkins, 2010). Hence, male chimpanzees may increase the perceived effect of their pant hoots by first capturing the attention of nearby conspecifics using leaf clipping. Regardless, it remains that leaf clipping in combination with pant hoots appears to be a relatively rare behaviour, since the majority of male pant hoots in this community were produced without leaf clipping. Detailed field research into this behaviour, and those similar to leaf clipping, is needed to help disentangle some of the mechanisms proposed above.

## Conclusion

Based on our findings, we show that many acoustic parameters of the male chimpanzee pant hoot were significantly impacted by social instability during an alpha takeover and the occurrence of leaf clipping. Specifically, pant hoots accompanied by leaf clipping were longer, had more call units and drum beats, and lower F0 and pF in the build-up phase. Yet during the period of instability, pant hoots were generally shorter, had fewer call units and drum beats and higher F0 and pF in the build-up phase. Since all males of this chimpanzee community have now been observed to leaf clip, including those that were too young at the time of this study (Taï Chimpanzee Project, unpublished data, 2012–2018), the leaf clipping behaviour appears to continue to maintain itself as a socio-cultural trait in this community. Although in this study we did not examine specific aspects of social learning that presumably enabled the successful transmission of the leaf clipping behaviour in this group, previous chimpanzee research has shown that social learning is at least in part responsible for the spread and maintenance of socio-cultural behaviours (Leeuwen et al., 2012). Further research is needed to assess whether the results reported here are indicative of a general phenomenon whenever leaf clipping and pant hooting co-occur, or if it is limited to this chimpanzee group, and which proximate mechanisms are responsible for the observed acoustic variation.

##  Supplemental Information

10.7717/peerj.5079/supp-1 Table S1 Full versus null model comparisons did not change significantly when behavioural activity of the pant hoots were included in the18 GLMMs as an additional control variable with random slopes Consequently, significance of the individual test predictors was also verified to not change; however, due to model instability for some estimates, behavioural activity (travel, rest or feeding) was removed as a control from the final models reported in the main manuscript (Fig. 2, Tables 3, 4).

10.7717/peerj.5079/supp-2 Table S2 Mean and associated standard error for all 18 acoustic variables measured from male chimpanzee pant hoots for the three periods of instability and when leaf clipping occurred and did not occur

10.7717/peerj.5079/supp-3 Figure S1 Acoustic parameters of the introduction phase of the pant hoot that changed with respect to male chimpanzee rank. The darker the colour the more data points present (*N* = 173 for each plot) Male ranks have been standardized between a value of 0 and 1 due to the variable number of males in the group during the study period.

10.7717/peerj.5079/supp-4 Data S1 Spectral and temporal parameters measured from each chimpanzee pant hoot vocalization

10.7717/peerj.5079/supp-5 Data S2 Daily focal follow data of the five chimpanzee males used to calculate pant hoot and aggression rates

10.7717/peerj.5079/supp-6 Supplemental Information 1 Taï male chimpanzee leaf clips with a pant hoot Freddy, from the neighbouring East group chimpanzees of the Taï forest, is observed leaf clipping before emitting a pant hoot with buttress drumming.

## Additional Information and Declarations

### Field Study Permissions

The following information was supplied relating to field study approvals (i.e., approving body and any reference numbers): The Ministère de la Recherche Scientifique, the Ministère de l’Environnement et des Eaux et Forêts and the Office Ivorien des Parcs et Reserves in Côte d’Ivoire provided full approval for the purely non-invasive and observational research in this study (Ref: 11/MINEF/OIPR/DT/CAT).

### Data Availability

The following information was supplied regarding data availability: Data can be found in the Supplemental Information.
