# Supplementary material for: Re-emergence of the leaf clip gesture during an alpha takeover affects variation in male chimpanzee loud calls
Source: PeerJ. 2018 Jun 28;6:e5079. doi: 10.7717/peerj.5079 (PMC6026532; doi:10.7717/peerj.5079)
Supplement: Table S1 — Consequently, significance of the individual test predictors was also verified to not change; however, due to model instability for some estimates, behavioural activity (travel, rest or feeding) was removed as a control from the final models reported in the main manuscript (Fig. 2, Tables 3, 4). [file peerj-06-5079-s001.docx]

| Response variable | Full vs Null Model Comparison with Behavioural Activity included as a control |
| --- | --- |
| total duration (s) | χ^2^=16.57, df=4, P=0.0023 |
| # calls in the introduction | χ^2^=10.81, df=4, P=0.029 |
| introduction duration (s) | χ^2^=9.22, df=4, P=0.056 |
| F0 of last call of the introduction (Hz) | χ^2^=7.72, df=4, P=0.11 |
| duration of the last call of the introduction (s) | χ^2^=4.44, df=4, P=0.35 |
| pF of the last call of the introduction (Hz) | χ^2^=7.16, df=4, P=0.13 |
| # of voiced calls in the build-up | χ^2^=13.61, df=4, P=0.0087 |
| duration of the build-up (s) | χ^2^=1.76, df=4, P=0.78 |
| F0 of the middle call of the build-up (Hz) | χ^2^=16.00, df=4, P=0.003 |
| duration of the middle call of the build-up | χ^2^=14.47, df=4, P=0.0059 |
| pF of the middle call of the build-up (Hz) | χ^2^=13.69, df=4, P=0.0084 |
| # of elements in the climax | χ^2^=29.01, df=4, P=0.00001 |
| duration of the climax (s) | χ^2^=21.91, df=4, P=0.00024 |
| F0 of the highest call of the climax (Hz) | χ^2^=6.04, df=4, P=0.20 |
| duration of the highest call of the climax (s) | χ^2^=1.76, df=4, P=0.78 |
| pF of the highest call of the climax (Hz) | χ^2^=11.36, df=4, P=0.027 |
| duration of drumming (s) | χ^2^=31.24, df=4, P<0.00001 |
| # of drum beats | χ^2^=24.95, df=4, P=0.0001 |

*Transformation of the response, sample size, error structure and link functions were all identical to Table 3 in the main manuscript.
