# Supplementary material for: Re-emergence of the leaf clip gesture during an alpha takeover affects variation in male chimpanzee loud calls
Source: PeerJ. 2018 Jun 28;6:e5079. doi: 10.7717/peerj.5079 (PMC6026532; doi:10.7717/peerj.5079)
Supplement: Table S2 [file peerj-06-5079-s002.docx]

|  | Acoustic Parameter | Period of Instability | | | Leaf clipping | |
| --- | --- | --- | --- | --- | --- | --- |
|  |  | Before | During | After | NO | YES |
|  | total duration (s) | 7.71 ± 0.49 | 6.39 ± 0.37 | 7.19 ± 0.24 | 6.66 ± 0.17 | 9.58 ± 0.74 |
| **INTRODUCTION** | # calls in the introduction | 3.04 ± 0.38 | 3.65 ± 0.44 | 4.03 ± 0.26 | 3.48 ± 0.21 | 5.42 ± 0.51 |
|  | introduction duration (s) | 1.92 ± 0.3 | 2.82 ± 0.36 | 3.02 ± 0.24 | 2.49 ± 0.16 | 4.56 ± 0.65 |
|  | F0 of last call of the  introduction (Hz) | 401 ± 32.0 | 383 ± 11.3 | 404 ± 8.44 | 393 ± 8.17 | 422 ± 17.0 |
|  | duration of the last call of the introduction (s) | 0.30 ± 0.03 | 0.43 ± 0.04 | 0.42 ± 0.02 | 0.39 ± 0.02 | 0.46 ± 0.03 |
|  | pF of the last call of the  introduction (Hz) | 577 ± 92.6 | 466 ± 32.4 | 506 ± 26.0 | 486 ± 23.4 | 610 ± 60.3 |
| **BUILD-UP** | # of voiced calls in the build-up | 10.4 ± 0.82 | 7.51± 0.66 | 7.99 ± 0.39 | 8.18 ± 0.33 | 8.46 ± 1.00 |
|  | duration of the build-up (s) | 3.46 ± 0.25 | 3.10 ± 0.26 | 3.09 ± 0.13 | 3.17 ± 0.12 | 2.98 ± 0.27 |
|  | F0 of the middle call of the  build-up (Hz) | 296 ± 11.8 | 369 ± 15.9 | 355 ± 7.78 | 355 ± 7.41 | 314 ± 8.55 |
|  | duration of the middle call of the build-up (s) | 0.141± 0.004 | 0.214 ± 0.022 | 0.156 ± 0.007 | 0.176 ± 0.008 | 0.122 ± 0.007 |
|  | pF of the middle call of the  build-up (Hz) | 316 ± 31.2 | 389 ± 23.5 | 379 ± 16.4 | 381 ± 14.1 | 314 ± 8.55 |
| **CLIMAX** | # of elements in the climax | 9.43 ± 1.03 | 4.09 ± 0.51 | 5.92 ± 0.44 | 5.52 ± 0.37 | 8.96 ± 0.96 |
|  | duration of the climax (s) | 2.44 ± 0.26 | 1.35 ± 0.13 | 1.62 ± 0.09 | 1.62 ± 0.08 | 2.05 ± 0.24 |
|  | F0 of the highest call of the  climax (Hz) | 1221 ± 95.1 | 1097 ± 67.1 | 1114 ± 40.6 | 1109 ± 35.6 | 1271 ± 82.6 |
|  | duration of the highest call of the climax (s) | 0.49 ± 0.04 | 0.57 ± 0.04 | 0.55 ± 0.02 | 0.55 ± 0.02 | 0.51 ± 0.06 |
|  | pF of the highest call of the  climax (Hz) | 1848 ± 232 | 1532 ± 153 | 1447 ± 104 | 1546 ± 90.2 | 1474 ± 174 |
|  | duration of drumming (s)* | 1.55 ± 0.23 | 0.46 ± 0.10 | 0.85 ± 0.09 | 0.77 ± 0.08 | 1.28 ± 0.21 |
|  | # of drum beats* | 7.07 ± 0.92 | 2.23 ± 0.40 | 4.42 ± 0.42 | 3.75 ± 0.33 | 6.93 ± 0.71 |

^*drumming could start in the build-up or climax but usually occurred solely in the climax phase^
