# Supplementary figures and images for: Re-emergence of the leaf clip gesture during an alpha takeover affects variation in male chimpanzee loud calls
Source: PeerJ. 2018 Jun 28;6:e5079. doi: 10.7717/peerj.5079 (PMC6026532; doi:10.7717/peerj.5079)

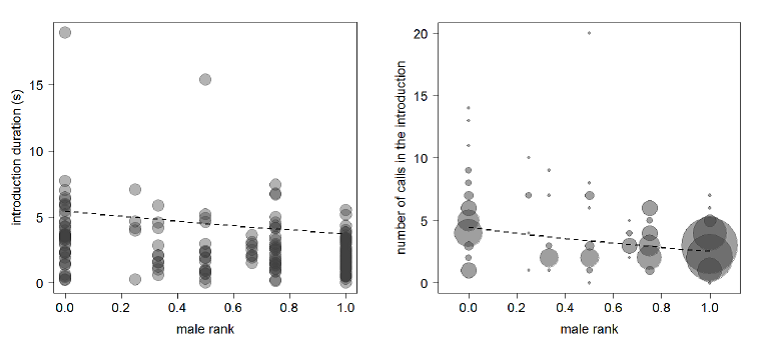

Supplement: Figure S1 — Male ranks have been standardized between a value of 0 and 1 due to the variable number of males in the group during the study period. [file peerj-06-5079-s003.png]
